# The effect of nicotine patches on craving in the brain: A functional MRI study on heavy smokers

**DOI:** 10.1097/MD.0000000000012415

**Published:** 2018-09-28

**Authors:** Keliane Liberman, Peter Van Schuerbeek, Sarah Herremans, Marc Meysman, Johan De Mey, Nico Buls

**Affiliations:** a Gerontology Department, Vrije Universiteit Brussel (VUB); b Departement of Radiology; c Department of Psychiatry; d Department of Pneumology, Universitair Ziekenhis Brussel (UZ Brussel), Brussels, Belgium.

**Keywords:** addiction, brain, cigarettes, craving, functional MRI, nicotine patch

## Abstract

**Background:**

Smoking is a common phenomenon and kills over 6 million people every year. Many smokers try to quit smoking by using nicotine replacement therapy (NRT). Most of the time, relapse occurs in less than six months after finishing the program of NRT. We performed a single blinded study in which our aim was to figure out what the effect of the nicotine patch is on craving in the brain of smokers deprived from smoking.

**Methods:**

Five heavy smokers (Fagerström Test for Nicotine Dependence ≥4) underwent a functional magnetic resonance imaging (fMRI) in 4 random conditions: smoking (S); smoking deprivation (SD); SD combined with a NP (SD + NP); SD combined with a placebo patch (SD + PP). Visual stimulation provoked craving in block design by randomly displaying images of smoking related scenes. After image preprocessing, a fixed-effect analysis was performed to compare average group activations. The Questionnaire for Smoking Urges (QSU) was obtained before and after each scan.

**Results:**

The fMRI results showed higher activation in areas involved in craving in S compared with SD + NP, SD + PP, and SD. In the SD + NP, limbic circuit and attention area were higher activated compared with SD and SD + PP. The SD + PP and SD showed higher activation in the frontal cortex and limbic system compared with S and SD + NP. Nonsmokers showed higher limbic activation compared with SD.

The QSU increased significantly after the fMRI experiment in S (*P* = .036).

The SD had higher QSU scores compared with the S before (*P* = .002), and also after (*P* = .022) the fMRI experiment. The NP showed lower scores than the SD before the experiment (*P* = .046).

**Conclusion:**

The fMRI experiment revealed lower activity in areas associated with attention when subjects were nicotine deprived (SD + PP and SD). Areas involved with craving showed less activity when nicotine is present (S and SD + NP). The QSU showed a significant difference between SD and when nicotine is present (S and SD + NP).

## 1 Implications

In this study, we used functional magnetic resonance imaging (fMRI) scans with visual stimuli to investigate the effect that transdermal nicotine patches have on brain activity of smokers. Compared with a group of smokers deprived from smoking and to a group wearing a placebo patch, people wearing nicotine patches showed reduced activity in areas of the brain that are associated with reward and craving, such as the limbic regions (putamen, lentiform nucleus, parahippocampal gyrus). The results of this study help to better comprehend the impact of nicotine replacement therapy on craving in the brain, and may contribute to understanding their effectiveness.

## 2 Introduction

Globally, smoking causes major health problems. Cigarette smoking increases risks for diseases such as coronary heart diseases, strokes, and lung cancer. When quitting, these risks drop significantly.^[1]^

The effects of nicotine can be divided into pharmacological and psychodynamic effects. Pharmacological effects include a higher heart rate, increased stroke volume, and a higher use of oxygen. Under psychodynamic effects, the most common are euphoria, increased alertness, and feeling of relaxation.^[2]^ A third characteristic of nicotine is the addiction, defined by American Society of Addiction Medicine as “a primary, chronic disease of brain reward, motivation, and related circuitry.”^[3]^

The addiction characteristic is the main reason relapse occurs when trying to quit smoking. Craving has a major role in relapse.^[4]^ Nicotine binds on the presynaptic receptors of glutamate and postsynaptic receptors of dopamine neurons in the ventral tegmental area (VTA), leading to dopamine release in the nucleus accumbens (NAS) and the prefrontal cortex (PFC), known as the reward circuit. Due to the up-regulation of the dopamine and nicotine receptors, there is a constant need of nicotine in the brain, often leading to relapse when trying to quit.^[4]^

The intention of nicotine replacement therapy (NRT) is to reduce the craving to smoke, and to prevent withdrawal symptoms caused by a lack of nicotine in the brain. Nicotine patches (NPs) release nicotine transdermal immediately into the bloodstream with a constant rate, avoiding first-pass-effect and maintaining a more stable, longer-term nicotine level throughout the body and brain.^[5]^ The patches show highest effectiveness in quitting rates compared with other NRT monotherapy.^[6,7]^ The evaluation of the patches is important to the field of NRT because smokers who try to quit commonly use it.

Functional MRI is a noninvasive (no ionising radiation, no contrast agent needed) technique that allows to study the function of specific brain regions in high detail by performing specific cognitive tasks, such as watching pictures.^[8]^

During a task, vasodilatation occurs and the blood flow increases in the brain region that is activated due to the higher demand of oxygen in that region.^[9]^ During fMRI, regions with more deoxygenated hemoglobin, which causes inhomogeneity in the magnetic field, will give a decrease in the obtained signal (proportional to the neural activity) than regions with oxygenated hemoglobin. This is also known as the Blood Oxygenation Level Dependent (BOLD) effect.^[10]^

In this single-blinded randomized controlled study, the effect of NP on craving in the brain was assessed by fMRI. In addition, the questionnaire for smoking urges (QSU) was conducted to evaluate craving before and after the fMRI experiment.

Nonsmokers were included to evaluate whether the cue exposure was salient for our smokers. The major aim was to investigate the effect of NP on craving in the brain by comparing 4 different conditions in our smoking participants: continuously smoking (S), smoking deprivation with nicotine patch (SD + NP), smoking deprivation with placebo patch (SD + PP), and smoking deprivation with no patch (SD). We hypothesize that when an NRT is applied, craving will decrease and that there will be an impact on the BOLD signal in the reward area of the brain.

## 3 Material and methods

### 3.1 Subjects

Healthy smoking volunteers aged >18 years were included. Inclusion criteria were: a score ≥4 on the Fagerström Test for Nicotine Dependence (FTND),^[11]^ in good general health, and to be able to undergo an MRI scan. All participants were recruited through flyers and e-mail. Participants who consumed nicotine in any other form than cigarettes, had psychiatric or neurologic disorders, pregnant, or engaged in smoking cessation treatment were excluded. Other exclusion criteria were the use of medication (contraception was allowed), dependency to alcohol or other drugs, medical illness, or allergy to any compound of the patches.

**Figure 1:**
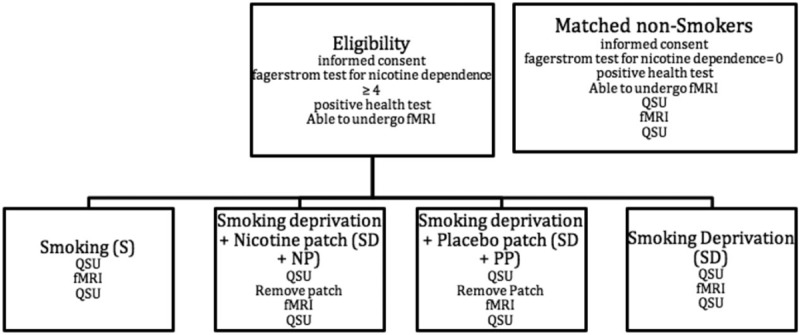
Flowchart of the experimental procedures for each participant. For each condition, the same routine took place, where an anatomical scan preceded the fMRI experiment. The craving test was taken before and after the fMRI scans. fMRI = functional magnetic resonance imaging, QSU = Questionnaire for Smoking Urges.

To investigate the effects of the NP on the craving and neural activity of specific brain regions, we defined 4 conditions for the smoking group (Fig. 1): S—where smoking behavior was maintained until entering the hospital for the experimental fMRI; SD + NP—where participants were asked to quit smoking the evening before the day of scanning, and applied a nicotine patch; SD + PP—where the participants quit smoking and applied a placebo patch; SD—participants quit smoking the evening prior scanning day and did not place any patch. The order of conditions was randomized for each participant.

As a control, healthy nonsmoking volunteers matched for sex, age, and left-right-handedness were included to reduce variability between participants. Exclusion criteria for nonsmokers were the same as for smokers, with exception of an FTND score equal to 0 (Fig. 1).

Participants provided a written informed consent. Participants were compensated for completing the study. Approval was granted from the ethics committee of the UZ Brussel (B.U.N. 143201421919).

### 3.2 Experimental set-up

Transdermal de-identified NP (NiQuitin Clear, GlaxoSmithKline, Moon Township, PA) and PP were used. PPs were self-manufactured by using plastic foil and placing them into band aids, ensuring they look exactly like the de-identified NP. NP of 21 mg/24 h were to alleviate withdrawal symptoms during smoking cessation. Participants were blinded to whether they received a NP (21 mg nicotine) or PP (0 mg nicotine). Instructions were given to the volunteers to apply the patches on the upper left arm between 7:00 and 8:00 o’ clock in the morning of the day of the experiment. No alcohol was allowed the day of scanning, and coffee was prohibited at least 4 hours before scanning. Patches were removed before entering the scanner to avoid potential skin burns due to the metalized layers in the patches.^[12,13]^

The standard Brief Questionnaire for Smoking Urges (Brief-QSU),^[14]^ containing 10 statements as for example “I crave for a cigarette right now,” where participants were asked to answer on a scale of 0 (I do not agree at all) to 7 (I agree entirely), was conducted just before and immediately after scanning. This was done to validate our cue exposure and findings of the fMRI experiment. In all conditions, before the fMRI, an anatomical brain scan was made. Scanning procedures included 4 sequential scans in randomized order for smokers, with an interscan interval of a week to avoid a wearing off phenomenon. For nonsmokers, the study required only 1 visit where all same questionnaires were conducted and only 1 fMRI scan was performed (Fig. 1).

### 3.3 Image acquisition

We utilized a 3T Phillips scanner (Achieva, Philips Healthcare Best, the Netherlands), and BOLD responses were acquired. T1-weighted anatomical scans were performed using following sequence: matrix = 256 × 256, voxel dimension = 1 × 1 × 2 mm, field of view (FOV) = 240 mm.

Functional images were acquired through T2 spin echo EPI scans: TE = 70 ms, TR = 3000 ms, flip angle = 90 degrees, FOV = 230 mm, 31 slices, slice thickness = 3 mm, matrix = 128 × 128, 31 slices, ISI = 0.5 mm, voxel dimension = 1 × 1 × 4 mm.

### 3.4 fMRI experiment

**Figure 2:**
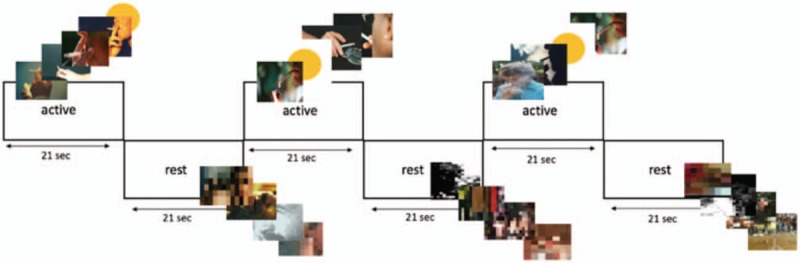
Design of the visual task used during the fMRI experiment. fMRI = functional magnetic resonance imaging.

The fMRI experiment was programmed and run by E-prime (Psychology Software Tools, Pittsburgh, PA). Foam sponges were used to minimize participants’ head movement. Images appeared in blocks of 21 seconds. Blocks of neutral images alternated 7 blocks of stimuli. Pictures of smoking persons, lit up cigarette, and hands holding cigarettes from the International Smoking Image Series (ISIS)^[15]^ were used as active stimuli. These pictures were used and validated in previous studies.^[16,17]^ Blurred, smoothed pictures served as neutral pictures (Fig. 2). A yellow dot was randomly presented once during each stimulus block. When the dot appeared, participants were asked to press on the reaction button, ensuring participants’ attention to the images. Pictures each appeared during 3 seconds, which implicates that there was a total of 6 different images from the ISIS and 1 yellow dot during each active block, and 7 neutral pictures during rest conditions. Two versions of the block design were used to ensure participants did not know or remember when the dot would appear during the active blocks.

### 3.5 Analysis of the fMRI data and statistics

All fMRI experiments resulted in 105 brain scans. Statistical Parametric Mapping (SPM8) (Wellcome Department of Cognitive Neurology, London, UK) running in MATLAB (The Mathworks, Inc., Natick, MA) was used for image analysis. All data were collected and stored on the local server of the radiology department of the UZ Brussel.

**Figure 3:**
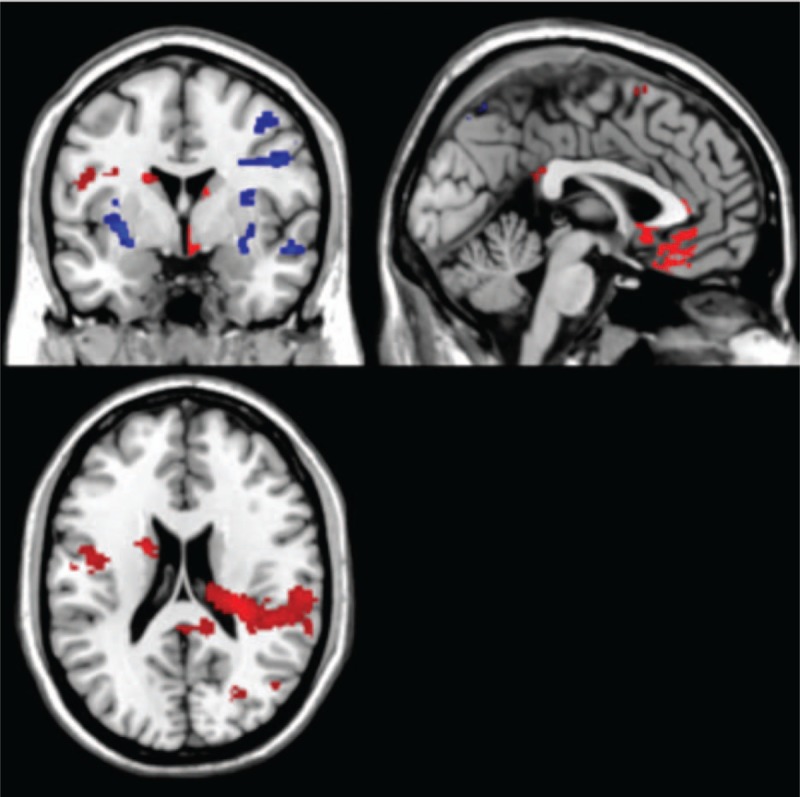
The figure displays the difference image when comparing nonsmokers and smokers in a SD condition. The regions shown on the image show where there was a higher activation in 1 condition compared with the other one. Threshold: *P* ≤ .005; voxel size ≥50 voxels; red = nonsmoker; blue = SD. Nonsmokers show higher activation in the frontal cortex and limbic system. Deprived smokers showed higher frontal and limbic system activation in other specific regions.

To validate our cue exposure task, the scans from the nonsmokers were compared with the scans from the smokers taken during the SD condition (Fig. 3). For this, we analyzed the individual fMRI scans using the general linear modeling (GLM) approach. As regressors in the model we used the timings of the active condition and the yellow dots, both convolved with the hemodynamic response function (HRF). The 6 motion parameters and a constant to model the signal offset were added to the model as regressors of no interest. For each individual scan, the contrast maps ‘Smoking > neutral,’ representing in each voxel the increase or decrease in neural activity during the active task condition, were derived from the fitting results. Finally, these contrast maps were entered into a 2-sample *t* test (Table 1).

**Table 1:**
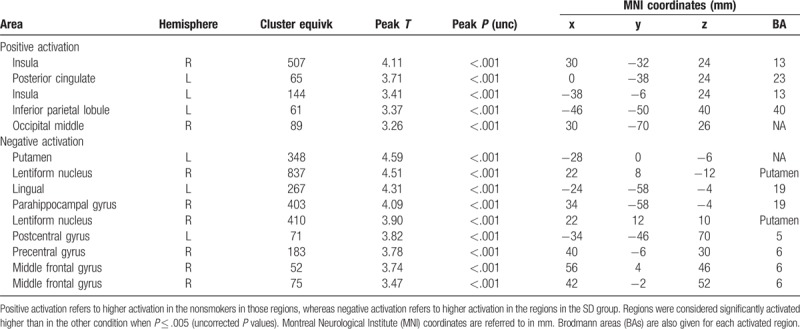
Comparison of the differences in activation between nonsmoker group and the SD condition in the smoker group.

**Figure 4:**
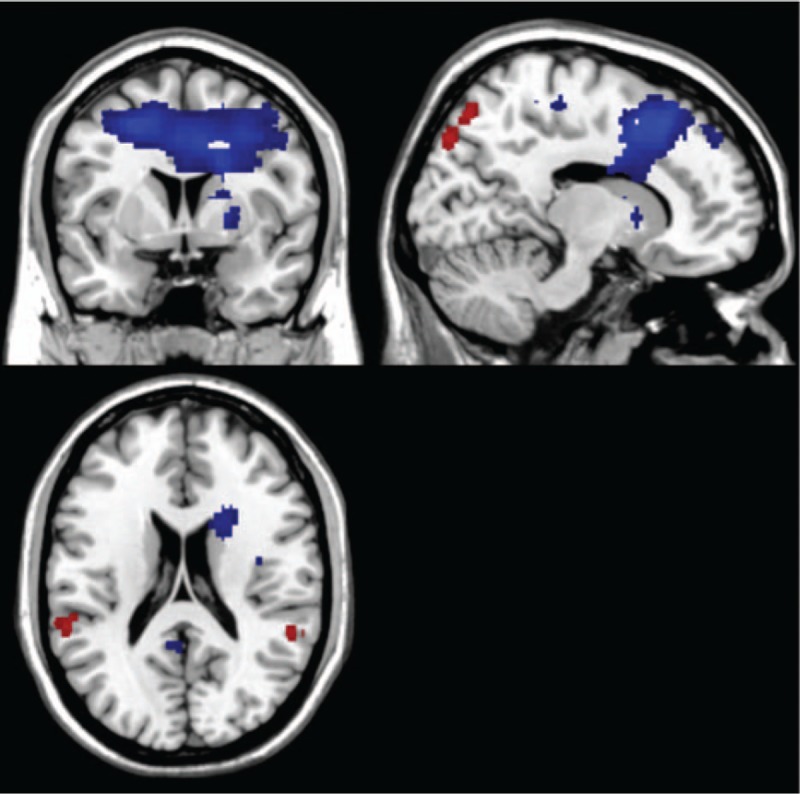
Comparison between the nicotine and placebo patch in smokers. The regions shown on the image show where there was a higher activation in 1 condition compared with the other one. Threshold: *P* ≤ .005; voxel size ≥50 voxels; red = SD + NP; blue = SD + PP. The placebo patch showed higher frontal activation compared to the nicotine patch, which showed higher activation in attention areas, temporal and parietal.

**Figure 5:**
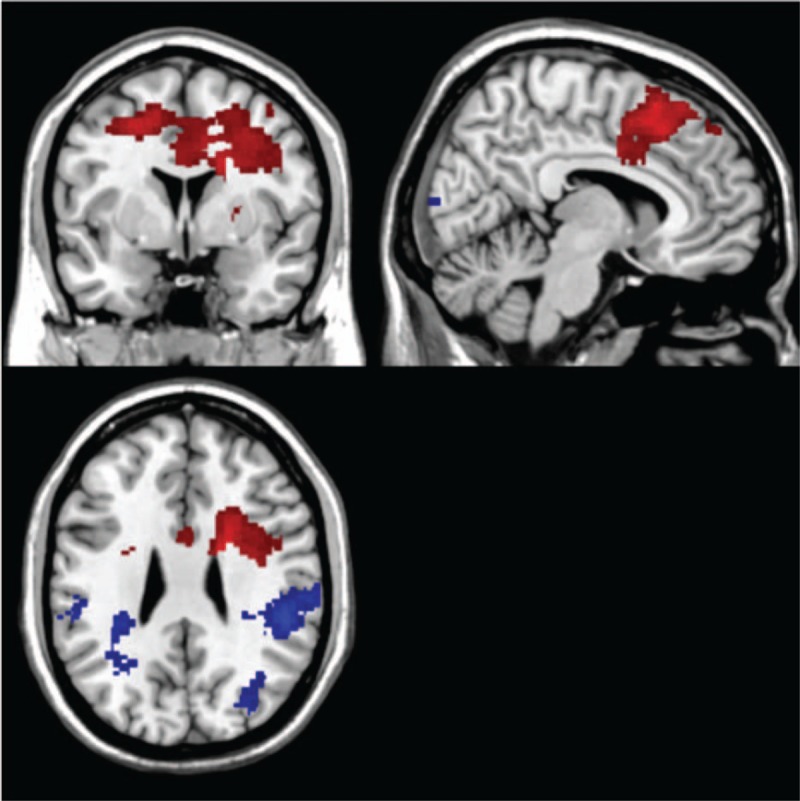
The differences between the SD + NP condition and the SD condition. The regions shown on the image show where there was a higher activation in 1 condition compared with the other one. Threshold: *P* ≤ .005; voxel size ≥50 voxels; red = SD; blue = SD + NP. We mainly notice higher frontal activation and also limbic activity. With the nicotine patch, attention areas and limbic system showed higher activation.

**Figure 6:**
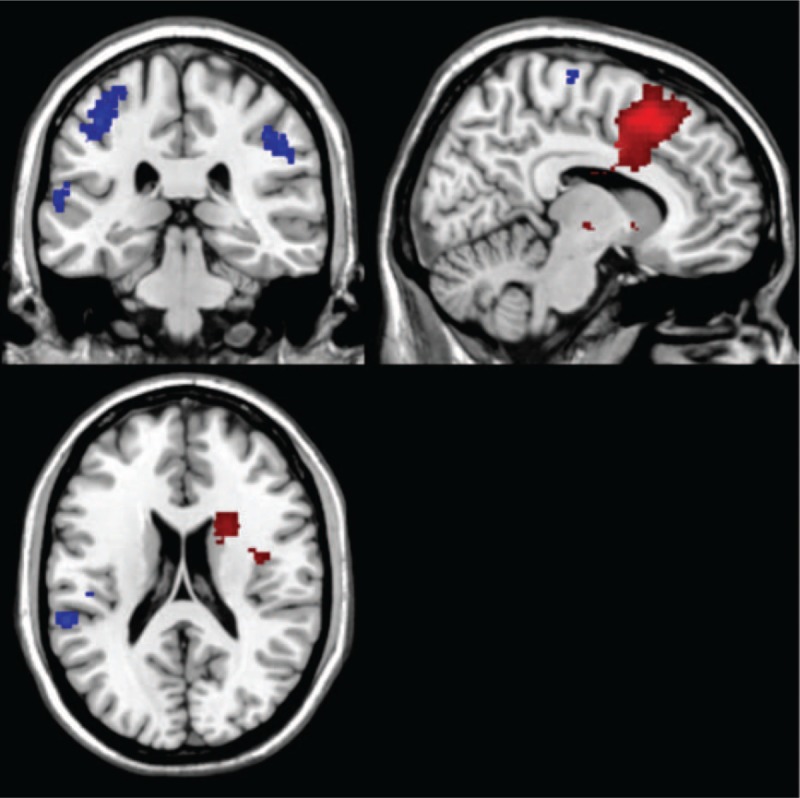
Comparison between S condition and the SD + NP condition. The regions shown on the image show where there was a higher activation in 1 condition compared with the other one. Threshold: *P* ≤ .005; voxel size ≥50 voxels; red = S; blue = SD + NP. In the S condition, we noticed higher activation in the frontal cortex. In the SD + NP condition, frontal and limbic system were activated higher.

Secondly, we performed a fixed-effect analysis to compare the various smoking conditions within the smokers group. For this, all fMRI scans were entered in 1 big GLM as multiple subjects and multiple sessions. For each scan, the model consisted of the timings of the active condition and the yellow dots, both convolved with the HRF, as regressors, and the 6 motion parameters and a constant as regressors of no interest. Based on the fitting results for the active task condition regressors, *t* tests were performed to test the significance of the contrasts ‘NP versus PP,’ ‘NP versus SD,’ and ‘NP versus S’ (Figs. 4–6, Tables 2–4).

**Table 2:**
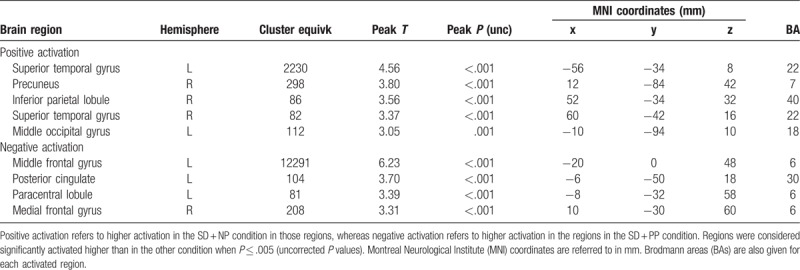
Comparison of the differences in activation between the SD + NP condition and the SD + PP condition in the smoker group.

**Table 3:**
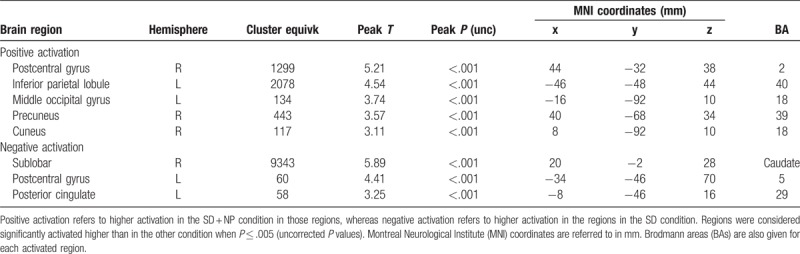
Comparson of the differences in activation between the SD + NP condition and the SD condition in the smoker group.

**Table 4:**
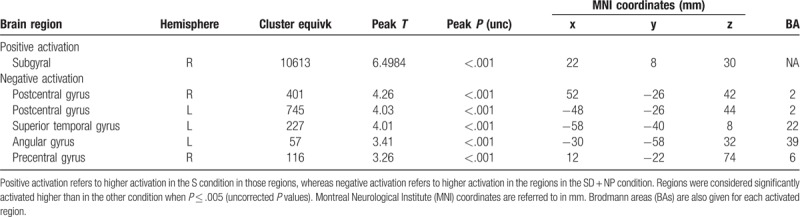
Comparison of the differences in activation between the S condition and the SD + NP condition in the smoker group.

To reduce the risk for false positive results, only clusters surviving a voxel significance *P* < .005 and a cluster extend threshold Ke >50 voxels were considered as significant. WFU pickatlas was used to convert obtained MNI coordinates into Brodmann areas (BAs).^[18]^

For comparison of the QSU scores, first, an overall repeated-measures analysis of variance (ANOVA) was performed. As post hoc, the paired *t* test was used to compare QSU scores before and after the fMRI experiment within a condition, and also comparison between conditions. Results with a *P* < .05 were considered significant.

## 4 Results

### 4.1 Sample characteristics

In all, 21 smokers were screened, of which 10 were excluded due to a too low FTND score; 4 because they were treated with pharmacological substances interfering with the experiment, resulting in 7 eligible participants. Two participants did not complete all conditions; therefore, the final sample consisted out of 5 participants (1 male and 4 females, aged 21.7 ± 3.8 years, FTND 5.2 ± 1.1). Five nonsmokers matched for age, sex, and left-right-handedness were also recruited. Baseline characteristics showed significant difference for the FTND score between groups (*P* < .001).

### 4.2 Craving scores

In our repeated-measures ANOVA, we found an overall significant interaction between the smoking conditions and before and after the fMRI experiment (*F* = 29.509, *P* = .033).

The craving scores of smokers in the S condition only showed significant increases after the fMRI experiment (*P* = .036). When wearing a patch (placebo and nicotine), no significant differences were found (Table 5).

**Table 5:**
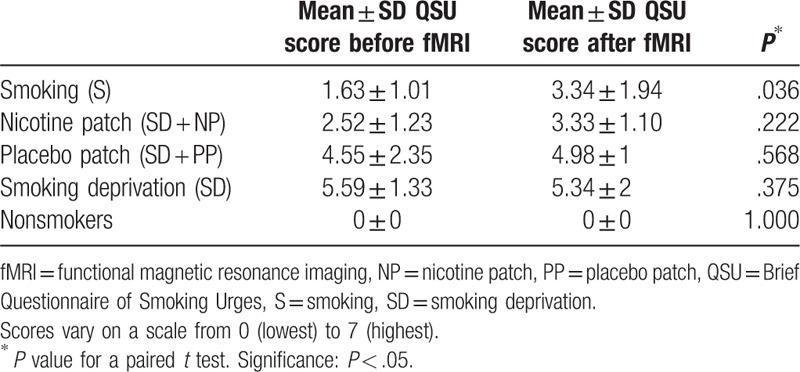
Mean craving scores for each separate condition before and after the experimental fMRI scan.

The QSU scores were compared between conditions, before and after the fMRI experiment (Table 6). There were significant differences between the S and SD group, before and after the fMRI experiment (*P* = .002 and *P* = .022, respectively). The SD group had higher QSU scores compared with the S group. The NP showed lower scores than the SD before the experiment (*P* = .046).

**Table 6:**
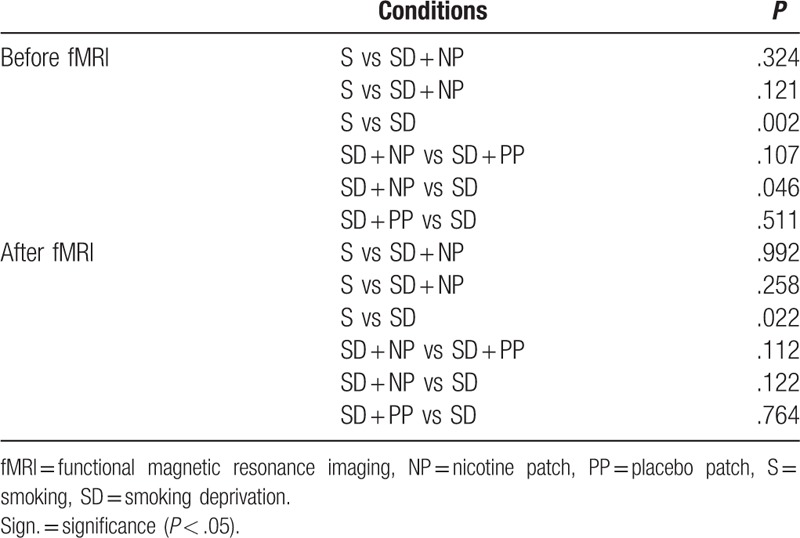
Comparison between craving scores between 2 conditions before the fMRI experiment and after the fMRI experiment.

### 4.3 fMRI results

We compared the NP condition with the other 3 conditions in the smokers group: S, SD, and SD + NP. Detailed tables including significantly activated brain areas, hemispheres, cluster size, MNI coordinates, BAs, T-contrast, and *P* values are shown in Tables 1–4.

#### 4.3.1 Nonsmokers compared with smoking deprivation

Nonsmokers showed higher activation in the insula (BA 13), posterior cingulate (BA 23), inferior parietal lobule (BA 40), and middle occipital gyrus.

Smokers in the SD condition showed higher activation in the putamen, lentiform nucleus, lingual gyrus (BA 19), parahippocampal gyrus (BA 19), precentral gyrus (BA 6), and middle frontal gyrus (BA 6) (Fig. 3, Table 1).

#### 4.3.2 Nicotine patch compared with placebo patch

When comparing the nicotine withj the placebo patch, higher activation during the SD + NP was seen in the superior temporal gyrus (BA 22), inferior parietal lobule (BA 40), precuneus (BA 7), and middle occipital gyrus (BA 18). The placebo patch, on the contrary, showed higher activity in the middle and medial frontal gyrus (BA 6), posterior cingulate (BA 30), and paracentral lobule (BA 6) (Fig. 4, Table 2).

#### 4.3.3 Nicotine patch compared with smoking deprivation

Comparing the SD + NP with SD, we noticed higher activation sublobar, postcentral gyrus (BA 5) and posterior cingulate (BA 29) during smoking deprivation. During the SD + NP condition, we noticed higher activation in the following regions: postcentral gyrus (BA 2), inferior parietal lobule (BA 40), middle occipital gyrus (BA 18), cuneus (BA 18), and precuneus (BA 39) (Fig. 5, Table 3).

#### 4.3.4 Nicotine patch compared with smoking

Comparing the S condition with the SD + NP condition, we noticed higher activation in the subgyral during the smoking condition.

When applying the NP, the pre and postcentral gyrus (respectively, BA 6 and BA 2), superior temporal gyrus (BA 22), and angular gyrus (BA 39) were activated higher (Fig. 6, Table 4).

## 5 Discussion

The aim of this study was to investigate the effect of NP on craving in the brain through fMRI. In addition, the QSU was conducted to evaluate craving to smoke a cigarette before and after the fMRI experiment.

### 5.1 QSU

In line with our hypothesis, our QSU results showed that craving was high in smokers deprived from smoking. A significant increase was observed in the S condition after applying our visual cue exposure. Between conditions, there were significant differences between the S and SD group before, and also after the fMRI experiment. As we expected, the SD group had higher QSU scores compared with the S group. The NP showed lower scores than the SD before the experiment.

Craving is generally high in smokers, especially when the nicotine level decreases after smoking their last cigarette. As in our study, Thewissen et al^[19]^ showed that smokers, exposed to smoking cues, had a lower initial urge to smoke than after the experiment.

In the study performed by Bradstreet et al, the differences in abstinence durations were investigated through fMRI and craving tests. They showed that craving increased when there was a short abstinence period of 2 days when presenting a cue-smoking stimulus.^[20]^

Tiffany et al compared cue-elicited craving when wearing a transdermal NP or a PP. Using the QSU, they showed that craving increased after a smoking abstinence of 6 hours, although wearing NP or a PP.^[21]^ The study performed by Bell et al investigated cognitive performances after smoking deprivation, and also evaluated craving scores using the QSU. Their results showed that craving was significantly increased when there was an 18 hours smoking deprivation, with no NRT administered.^[22]^

Taking all these studies together and comparing these to our results, we noticed similar results. This shows that visual stimulation tends to increase craving in our smoking participants.

### 5.2 fMRI imaging results

In this study, we compared the SD + NP with SD + PP, SD, and S condition. Because subjects who are wearing a NP are expected to have lowest craving levels compared with the subjects of the SD + PP/SD conditions, we hypothesized that these subjects have the lowest BOLD response in those areas regulating addiction and reward. The contrary should be true for those subjects in SD, because these are expected to have highest craving levels. Because placebo-treated subjects probably have more craving than those with a NP, we expected to see higher activity in the areas regulating addiction and reward during SD + PP compared with SD + NP.

Our results showed that compared with SD, nonsmokers revealed higher limbic system activation, more specifically, the insula and cingulate. This system is primarily involved in regulating emotions. In nonsmokers, this was possibly a negative emotion, as explained by Berridge and Kringelbach^[23]^ towards viewing cigarette stimuli, caused by the disgust and opposition towards cigarettes. This is also a possible effect caused by the thought of second-hand smoking, which negatively affects nonsmokers.^[21]^ The SD showed more activity in limbic regions, which indicates positive feelings and reward. Also, BA 6 was highly activated in the smokers group. This area is part of the frontal cortex, involved in planning of motor activity.^[24]^ In our case, this could reflect the mirror neurons involved in the perception of the physical act of smoking. They were possibly thinking of the motoric gesture involved in smoking a cigarette and therefore the motoric regions show higher activity. This suggests that the addiction and craving were triggered.

The lingual gyrus was also more activated in SD, which, according to studies, is involved in the incentive salience, or motivational “wanting” in response to the smoking cues.^[25]^

In line with our expectations, craving did not increase when subjects with a NP were confronted with the visual stimuli. This is in line with previous fMRI research where an attenuation of craving increase, which was triggered by smoking deprivation, could be observed when placing a NP.^[21]^ The study also demonstrated that when wearing a NP, craving decreased compared with the PP where an increase in craving was observed. They showed that abstinence and cue exposure are 2 separate and independent contributions to craving in smokers. This explains why craving will never disappear completely when using NP; the craving induced by abstinence will be dampened, but the craving induced by cue exposure will remain.^[21]^

The results of the NP compared with the SD + PP revealed activations in the regions we expected. In the placebo condition, frontal and limbic system were activated higher, possibly indicating higher salience attribution to the stimuli. Also BA 6 was activated during the SD + PP condition, indicating the preparation of smoking the next cigarette.

During the NP condition, activation of precuneus was higher, indicating increased attention. Also, the temporal gyrus was activated higher during the NP condition, which is associated with the perception of emotions.^[26]^

This is not in line with a study performed by Sweet et al, where they compared the NP with the PP in smoking-deprived smokers. Temporal and medial frontal gyri were deactivated during complete withdrawal. Another result was that when applying the PP, more individual variation in brain response was noticed, which they suggest comes from an inefficient neural processing due to the lack of nicotine and increased craving. During placebo, also regions of the default network, being the medial frontal and temporal cortex were deactivated.^[27]^

When we compared the S condition with the SD + NP condition, frontal regions were activated more during the S condition, suggesting craving increased due to the visual smoking cues. While wearing a NP, we expected to see changes in the BOLD response in brain areas involved in craving. This was indeed noticeable, especially regions associated with reward and craving were activated. During the NP condition, BA 6 was activated higher than in S condition, which suggests that craving was higher. This was not the case when smokers had just smoked and they were satiated with nicotine.

Stein et al performed an fMRI study on smokers to investigate which areas are activated more when nicotine is present. Their results confirmed that when nicotine is present in shortly deprived smokers, higher activity is noticed in the frontal cortex, nucleus accumbens, cingulated, and amygdala.^[28]^ Comparing these results with ours, we see a big cluster in the frontal cortex that is more activated during SD + NP compared with SD.

Knott et al^[29]^ used electroencephalography to investigate cue reactivity in smokers and also found that craving increased after the cue exposure, especially in regular smokers compared to light smokers.

Hughes^[30]^ showed that smokers remaining in an abstinent state or placebo condition showed lower attention to the cue presentation. Jorenby et al^[31]^ showed, by using questionnaires, that this effect can be reversed when applying transdermal NP. This was also noticed in our results, where the cuneus and precuneus showed more activation in the SD + NP compared with the SD condition, where these areas were deactivated.

Tiffany et al^[21]^ showed that craving was higher in the smoking deprived condition than when they received a NP. Also in our study, the NP condition attenuated craving. Higher activation was noticed during the deprivation condition in the posterior cingulate, which is involved in craving.

Lawrence et al proved that the transdermal NP condition improved cognitive tasks in smokers as visual attention, arousal, and motor activation. More specifically, regions as the parietal cortex, caudate, and thalamus were more activated while wearing a NP.^[32]^ This is in line with our results, where also the inferior parietal lobule was activated more while wearing the patch compared with SD + PP or SD condition. This region, part of the default network, is known to be associated with the perception of emotions, and for the interpretation of sensory information, here our visual cue.^[33]^

Li et al^[34]^ recently found that cigarette craving after hypnotic suggestion can be predicted by functional connectivity to related brain regions. Targeting these specific areas through transcranial magnetic stimulation should be investigated to reduce craving after smoking cessation.^[35]^

Smoking expectancy had an effect during cue-induced neural activation. Participants expecting to smoke immediately after the fMRI experiments showed more activation in attention, arousal (namely thalamus and cingulate) and cognitive control compared with smokers who were not allowed to smoke 4 hours after the fMRI scan. Assessing craving, no differences were noticed.^[36]^

Overall, we saw that the patch reduced activation in areas involved in craving compared with the placebo or deprivation condition. Activation in attention areas were more activated when nicotine was presented through the NP.

Some limitations of our work have to be considered. Although we obtained significant results, an important limitation is the relatively small sample size. However, other fMRI studies reported that with comparable low sample sizes, representative results can be obtained.^[17]^ Another limitation of the study is the lack of control whether participants actually refrained from smoking during the 3 nonsmoking conditions. To resolve such issues a carbon monoxide breath test or blood test could have been applied. Also, we were not able to verify when participants placed the NP, or if they respected the instructions conforming to the study protocol.

Our protocol also required an overnight abstinence in smokers. Though this was enough to decrease the nicotine level and increase craving, it remains a short-term abstinence. Follow-up of the craving on the following hours was not performed.

Our smoker group expected to smoke immediately after the fMRI scan, which might interfere with the reality of smokers seeking to quit.

The evaluation of the patches is important to the field of NRT because smokers who try to quit commonly use it. Many people using NP relapse after a couple of months since the triggers in the environment remain. The results from our study suggest that activity in areas involved with craving is increased after the cue-induced craving.

This inquires research needs to be done concerning those triggers and cues inducing craving in abstinent smokers.

Earlier studies have investigated the effects of nicotine, nicotine abstinence, and/or nicotine administration using fMRI, and showed that there was activation in specific brain regions associated with attention, planning, and others related to addiction, dependence, and craving.^[37–39]^ However, studies show different, and sometimes, opposite results. To our knowledge, no study has yet been performed comparing all 4 conditions, and also comparing with nonsmokers.

Further research on the effect of new treatments (eg, electronic cigarettes) is necessary to better understand the effects of NRT's on craving in the brain.

## 6 Conclusions

Our fMRI results show that there is an increase in brain activity in areas involved in craving in the conditions in following order: smoking (S), deprivation with NP (SD + NP), deprivation with placebo patch (SD + PP), and deprivation (SD). When comparing conditions, there was a significant difference in craving between smoking deprivation and conditions where nicotine is present before the fMRI experiment. However, this difference disappeared in the SD + NP conditions after a visual cue exposure.

Importantly, from our imaging results, we can conclude that there is a lower brain activity in areas associated with attention in conditions where there is a lack of nicotine (the SD and SD + PP groups) after a smoking-related visual cue exposure. Areas involved with craving showed less brain activity in conditions where nicotine was present (S and SD + NP groups). We hypothesize that the NP helps to reduce craving compared with the placebo patch or smoking deprivation though craving never disappears.

## Author contributions

**Conceptualization:** Keliane Liberman, Peter Van Schuerbeek, Nico Buls.

**Data curation:** Keliane Liberman.

**Formal analysis:** Keliane Liberman, Peter Van Schuerbeek.

**Funding acquisition:** Johan De Mey.

**Investigation:** Keliane Liberman, Peter Van Schuerbeek, Nico Buls.

**Methodology:** Keliane Liberman, Peter Van Schuerbeek, Sarah Herremans, Nico Buls.

**Resources:** Johan De Mey, Nico Buls.

**Supervision:** Peter Van Schuerbeek, Nico Buls.

**Writing – original draft:** Keliane Liberman.

**Writing – review & editing:** Peter Van Schuerbeek, Sarah Herremans, Marc Meysman, Johan De Mey, Nico Buls.
